# Healthcare Predictions: unmasking the strengths and weaknesses of AI models for vitamin D deficiency assessment

**DOI:** 10.1016/j.jgeb.2026.100695

**Published:** 2026-04-24

**Authors:** Ahmad Al-Qerem, Ruzayn Quddoura, Abdulhakeem Issa, Asma Sbaih

**Affiliations:** a Department of Computer Science, Faculty of Information Technology, Zarqa University, Zarqa 13110, Jordan; b University of Business and Technology, Jeddah 21448, Saudi Arabia; c Engineering Department, Faculty of Engineering and Information Technology, Palestine Ahliya University, Bethlehem, Palestine

**Keywords:** Machine learning, vitamin D deficiency, Support vector machine, Random forest, Healthcare analytics, Explainability

## Abstract

This study evaluates eight machine-learning regression models for estimating serum vitamin D level as a support tool for vitamin D deficiency assessment. A cohort dataset of 100 individuals (dataset 132) was analyzed using Support Vector Machine (SVM), Random Forest (RF), Artificial Neural Network (ANN/MLP), Linear Regression (LR), Elastic Net (EN), Ridge Regression (RR), Lasso Regression (LSR), and RANSAC Regressor (RAN). Model performance was assessed over 30 repeated runs using mean absolute error (MAE), mean squared error (MSE), root mean squared error (RMSE), and coefficient of determination (R^2^). SVM yielded the strongest overall results (MAE = 1.841, MSE = 32.502, mean R^2^ = 0.9981), followed by RF (MAE = 7.571, MSE = 197.832, mean R^2^ = 0.9908). ANN showed intermediate performance (mean R^2^ = 0.8538), whereas RR was the weakest model (mean R^2^ = 0.4945). To address interpretability, the revised manuscript adds an explainability-oriented feature-attribute analysis derived from the correlation structure of the cohort. The strongest associations with vitamin D were gender (r = 0.64), hemoglobin (r = 0.47), age (r = 0.38), marital status (r = -0.35), and triglycerides (r = 0.32). These findings show that model choice substantially affects predictive performance and that nonlinear models, particularly SVM and RF, can support cost-conscious screening strategies for vitamin D deficiency assessment. Future work should validate the models on larger external cohorts and extend interpretability with model-specific explainability techniques.

## 1 Introduction

Vitamin D is essential for calcium homeostasis, bone metabolism, immune modulation, and several endocrine and cardiometabolic processes 1, 2. Despite its clinical importance, vitamin D deficiency remains highly prevalent and is influenced by lifestyle, diet, sunlight exposure, and comorbid conditions. Serum 25-hydroxyvitamin D is the standard laboratory marker for assessing vitamin D status, but universal biochemical testing is not always feasible, especially in resource-constrained settings Table 1Table 2.

**Table 1:** Representative studies on machine-learning-based vitamin D prediction and the research gap addressed in the present work.

**Study**	**Cohort/context**	**Models**	**Main finding**	**Key limitation/gap**
Garcia Carretero et al. [5]	1002 hypertensive patients	Comparative ML models with elastic-net-based reduction	Developed screening models with high sensitivity for vitamin D deficiency.	Single clinical population; hospital cohort limits generalizability.
Patino-Alonso et al. [6]	501 adults aged 35–75 years	Supervised learning using anthropometric variables	Predictive ability differed by sex and variable set.	Restricted predictor space; anthropometric features alone may omit laboratory markers.
Osmani and Ziaee [7]	CHB patients and healthy controls	Decision tree	Decision rules identified disease-specific risk factors and achieved high predictive accuracy.	Disease-specific cohort and limited methodological breadth.
Amiri et al. [8]	Maternal vitamin D deficiency setting	Optimized Bayesian network	Demonstrated the value of probabilistic modeling for obstetric risk assessment.	Focused on a narrow maternal/obstetric context.
Bechrouri et al. [9]	Vitamin D level prediction study	Statistical and machine-learning regressors	Reported variable performance across predictive models for serum vitamin D.	Stability across repeated runs and interpretability were not emphasized.
Guo et al. [10]	NHANES 2001–2018	Machine-learning prediction of deficiency	Showed the feasibility of population-scale vitamin D risk prediction from routinely collected variables.	Population survey setting differs from smaller cohort-based clinical prediction.

**Table 2:** Main hyperparameters used for the eight developed models.

**Model**	**Key settings used in this study**	**Purpose/rationale**
Support Vector Machine (SVM)	RBF kernel; C = 1,000,000; ε = 0.001	Captures nonlinear relations while preserving ε-insensitive regression.
Random Forest (RF)	1000 trees	Ensemble averaging reduces variance and improves stability.
Artificial Neural Network (ANN/MLP)	Hidden layers = (100, 100); max_iter = 1000	Learns nonlinear feature interactions through multilayer representations.
Linear Regression (LR)	Standard linear model after preprocessing	Provides an interpretable baseline for continuous vitamin D prediction.
Elastic Net (EN)	α = 0.1; l1_ratio = 0.9	Combines L1 and L2 regularization to balance shrinkage and selection.
Ridge Regression (RR)	α = 100; solver = cholesky; tol = 10^-4	Stabilizes linear estimates under multicollinearity.
Lasso Regression (LSR)	α = 0.1	Performs coefficient shrinkage and sparse feature selection.
RANSAC Regressor (RAN)	Base estimator = linear regression; max_trials = 100	Provides robustness against possible outliers through consensus fitting.

Artificial intelligence (AI) and machine learning (ML) are increasingly used in medicine to support screening, risk prediction, and clinical decision-making 3, 4. In this context, predictive modeling may provide an efficient way to estimate vitamin D level from routinely collected demographic and laboratory variables. Such models are not intended to replace biochemical measurement when it is clinically required; rather, they may help prioritize testing or support early assessment in settings where laboratory access is limited.

The current study focuses on regression-based prediction of continuous serum vitamin D level and interprets the results as a support tool for vitamin D deficiency assessment. This distinction is important because the outcome modeled in this manuscript is continuous rather than binary. Accordingly, the comparative evaluation relies on MAE, MSE, RMSE, and R^2^ instead of classification accuracy, sensitivity, or AUC.

The contributions of the revised manuscript are fourfold: (i) it presents a consistent comparison of eight regression models using the same cohort and repeated experimental design; (ii) it corrects the abstract and discussion so that the reported conclusions match the actual performance indicators; (iii) it adds an explainability-oriented feature-attribute analysis to identify the variables most strongly associated with vitamin D; and (iv) it provides mathematical formulations, detailed figure legends, and error bars for the performance figures.

### 1.1 Related work and research gap

Recent studies have shown growing interest in applying ML to vitamin D deficiency prediction. Garcia Carretero et al. [5] developed predictive models for deficiency screening in a hypertensive population and demonstrated the feasibility of ML-assisted prioritization of vitamin D testing. Patino-Alonso et al. [6] compared supervised approaches using anthropometric parameters and found that the predictive value of these variables differed by sex. Osmani and Ziaee [7] used a decision tree in chronic hepatitis B patients and healthy controls, showing that disease-specific biochemical markers can contribute to predictive modeling.

Other studies extended the problem to specific clinical scenarios. Amiri et al. [8] used an optimized Bayesian network for maternal vitamin D deficiency analysis, while Bechrouri et al. [9] compared statistical models for vitamin D level prediction and highlighted the variability of regression performance across methods. More recently, Guo et al. [10] demonstrated that population-scale ML models based on NHANES data can predict vitamin D deficiency using routinely collectable variables.

Although these studies are valuable, several gaps remain. First, the literature contains heterogeneous outcomes, study populations, and evaluation metrics, which makes direct comparison difficult. Second, explainability is often discussed only briefly, even though identification of influential features is clinically important. Third, repeated experimental assessment and uncertainty visualization are not consistently reported. The present study addresses these gaps by providing a repeated-run comparison, detailed metric reporting, error-bar visualization, and an explicit feature-attribute analysis grounded in the observed correlation structure of the cohort.

## 2 Materials and methods

### 2.1 Dataset source and cohort profile

The dataset labeled “132″ is the study-specific cohort dataset used in the present work rather than a public benchmark dataset. It contains 100 de-identified records and was used to investigate continuous serum vitamin D level in relation to demographic, laboratory, lipid, and genotype-related variables. The available variables include age, gender, marital status, hemoglobin, white blood cell count (WBC), total cholesterol (TC), triglycerides (TG), HDL, LDL, vitamin D, and genotype indicators (genotyper, GG, GT, TT, GTTT, G, and T).

The cohort covers ages 18–71 years (mean age = 33.4 years). The categorical distribution includes 68 males and 32 females, as well as 36 married and 64 unmarried participants. Because the study objective is regression-based assessment, vitamin D is treated as a continuous target variable rather than a categorical label.

### 2.2 Data preprocessing

During preprocessing, identifiers and redundant variables were removed to improve model usability and reduce noise. Columns such as “No.”, “vit D rec Apal”, and redundant genotype indicator columns that were not required for the final analyses were excluded. Categorical variables were encoded numerically, and the data were normalized before model training to place the predictors on comparable scales.

The workflow then split the cohort into training and testing subsets. To reduce dependence on a single random split, the training-and-evaluation cycle was repeated 30 times. The repeated-run strategy allows the average performance and standard deviation of each model to be reported, thereby giving a more stable view of predictive behavior.

### 2.3 MLViDPred framework

MLViDPred uses eight regression algorithms: SVM [11], RF [12], ANN/MLP [13], LR [18], RR [14], LSR [15], EN [16], and RANSAC Regressor [17]. The workflow consists of data ingestion, preprocessing, train–test splitting, model fitting, performance evaluation, and repeated execution over 30 runs. The goal is to identify the models that most reliably estimate serum vitamin D level in the available cohort.

Among the tested methods, SVM is used because it can model nonlinear relations through kernel transformation [11]. RF aggregates many decision trees to improve generalization and resist overfitting [12]. ANN/MLP captures nonlinear feature interactions through layered representations [13]. LR offers a transparent baseline [18], while RR, LSR, and EN provide regularized linear variants that are useful when predictors are correlated 14, 15, 16. RANSAC is included to examine the behavior of a robust regression strategy in the presence of possible outliers [17].

### 2.4 Mathematical formulation of the models

To improve methodological transparency, the main regression models used in MLViDPred are summarized below. Let x_i ∈ R^p denote the predictor vector for observation i and y_i denote the continuous serum vitamin D response.

(1) $$\mathrm{LinearRegression}:y\_i=\beta \_0+\Sigma \_{\left\{j=1\right\}}^{\{}p\}\beta \_jx\_\left\{ij\right\}+\varepsilon \_i$$

(2) $$\mathrm{Ridge}\;\text{Re}gression:\;\;\hat{\beta }=\;\arg\min\_\beta \;\left\{\;\left|\left|y\;-\;X\beta \right|\right|\_2^{\wedge }2\;+\;\lambda \left|\left|\beta \right|\right|\_2^{\wedge }2\;\right\}$$

(3) $$\mathrm{Lasso}\;\text{Re}gression:\;\;\hat{\beta }\;=\;\arg\min\_\beta \;\left\{\;\left|\left|y\;-\;X\beta \right|\right|\_2^{\wedge }2\;+\;\lambda \left|\left|\beta \right|\right|\_1\;\right\}$$

(4) $$\mathrm{Elastic}\;Net:\;\;\hat{\beta }\;=\;\arg\min\_\beta \;\left\{\;\left|\left|y\;-\;X\beta \right|\right|\_2^{\wedge }2\;+\;\lambda \left[\left(1-\alpha \right)/2\;\left|\left|\beta \right|\right|\_2^{\wedge }2\;+\;\alpha \left|\left|\beta \right|\right|\_1\right]\;\right\}$$

(5) $$\mathrm{SupportVectorRegression}:f\left(x\right)=w^{T}\varphi \left(x\right)+b,minimize1/2{\left\|w\right\|}^{2}+C\Sigma \_i\left(\xi \_i+\xi \_i^{*}\right)$$

(6) $$\mathrm{RandomForestRegression}:\hat{y}\left(x\right)=\left(1/B\right)\Sigma \_{\left\{b=1\right\}}^{\{}B\}T\_b\left(x\right)$$

(7) $$\mathrm{NeuralNetwork}:h^{(}l)=g\left(W^{(}l\right)h^{(}l-1)+b^{(}l)),\hat{y}=W^{(}L)h^{(}L-1)+b^{(}L)$$

(8) $$\mathrm{RANSAC}:\hat{\beta }*=argmax\_\beta |\{i:|y\_i-x\_i^{T}\beta |<\tau \}|$$

Equations (1), (2), (3), (4), (5), (6), (7), (8) show that the compared methods represent complementary modeling assumptions: transparent linear relations (LR), regularized linear shrinkage (RR, LSR, EN), nonlinear margin-based learning (SVM), ensemble learning (RF), multilayer nonlinear approximation (ANN), and outlier-robust consensus fitting (RANSAC).

### 2.5 Evaluation metrics and statistical analysis

Following standard regression-evaluation practice [19], model performance was quantified using MAE, MSE, RMSE, and R^2^. Lower MAE, MSE, and RMSE indicate smaller prediction error, while higher R^2^ indicates greater explained variance.

(9) $$\mathrm{MAE}=\left(1/n\right)\Sigma \_{\left\{i=1\right\}}^{\{}n\}|y\_i-\hat{y}\_i|$$

(10) $$\mathrm{MSE}=\left(1/n\right)\Sigma \_{\left\{i=1\right\}}^{\{}n\}{\left(y\_i-\hat{y}\_i\right)}^{2}$$

(11) $$\mathrm{RMSE}=sqrt\left(\left(1/n\right)\Sigma \_{\left\{i=1\right\}}^{\{}n\right\}{\left(y\_i-\hat{y}\_i\right)}^{2})$$

(12) $$R^{2}=1-\left[\Sigma \_i{\left(y\_i-\hat{y}\_i\right)}^{2}/\Sigma \_i{\left(y\_i-\overset{¯}{y}\right)}^{2}\right]$$

In addition, pairwise differences between algorithms were examined using the Wilcoxon signed-rank test [20]. This non-parametric test evaluates whether the paired performance scores of two models differ systematically across repeated runs. In the revised manuscript, p-values are reported together with an interpretation of whether the observed difference is statistically significant at the conventional 0.05 level.

## 3 Results and discussion

### 3.1 Correlation structure and explainability-oriented feature-attribute analysis

Reviewer comments requested an explainability-oriented analysis of the predictor attributes. To address this point, the revised manuscript now interprets the cohort correlation matrix as a transparent feature-attribute layer and ranks the variables according to their association with serum vitamin D. This analysis does not claim causality; rather, it helps identify which variables are most informative in the current cohort.

**Fig. 1:**
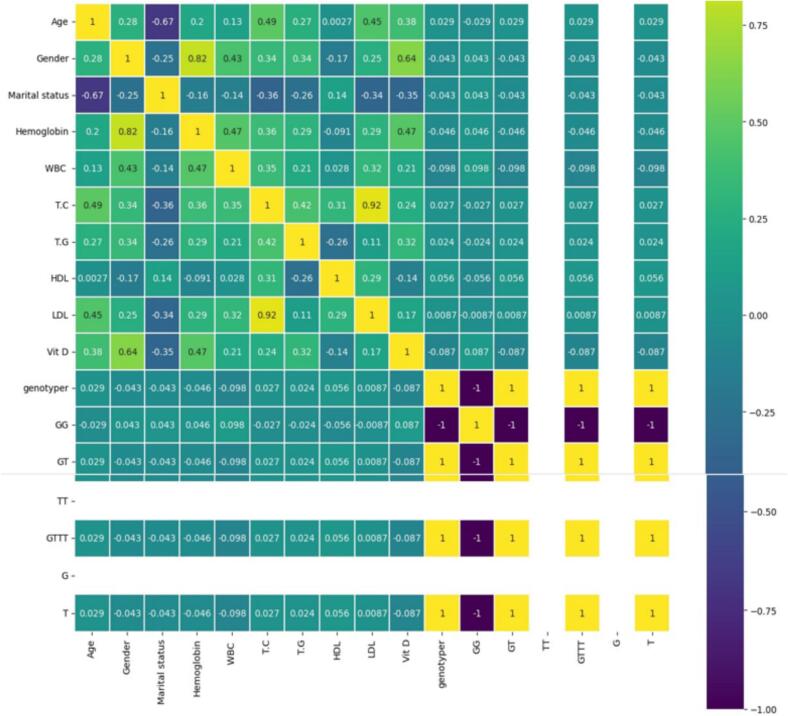
Correlation heatmap of the cohort after preprocessing. The color scale represents Pearson correlation coefficients between variables. The vitamin D column shows the strongest positive associations with gender (r = 0.64), hemoglobin (r = 0.47), age (r = 0.38), triglycerides (r = 0.32), total cholesterol (r = 0.24), WBC (r = 0.21), and LDL (r = 0.17), while marital status (r = -0.35) and HDL (r = -0.14) show negative associations. Genotype-related indicators exhibit only weak linear association with vitamin D in this cohort.

**Fig. 2:**
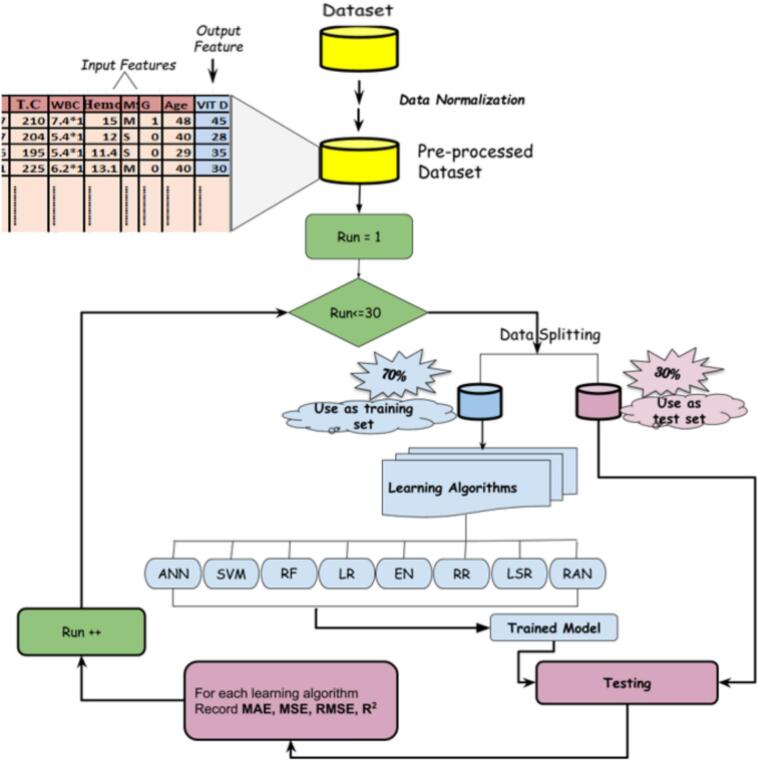
Stepwise MLViDPred workflow. The framework reads the cohort dataset, preprocesses and normalizes the input variables, splits the data into training and testing subsets, trains eight regression models, evaluates each model using MAE, MSE, RMSE, and R^2^, and repeats the process 30 times to improve the robustness of the comparative results.

As shown in Fig. 1 and Table 3, gender (r = 0.64) is the strongest observed predictor attribute in the current cohort, followed by hemoglobin (r = 0.47), age (r = 0.38), and marital status (r = -0.35). Among biochemical variables, triglycerides (r = 0.32), total cholesterol (r = 0.24), WBC (r = 0.21), and LDL (r = 0.17) contribute additional signal, whereas HDL shows a mild negative association (r = -0.14). Genotype indicators have only weak linear association with vitamin D in this dataset. These results provide a clinically interpretable complement to the predictive models by showing which variables are most strongly associated with the target outcome Fig. 2.Table 4.

**Table 3:** Ranking of the most influential cohort attributes based on their correlation with serum vitamin D.

**Predictor**	**Correlation with vitamin D**	**Interpretation for feature-attribute analysis**
Gender	0.64	Strongest positive association in the cohort and the most influential observed attribute.
Hemoglobin	0.47	Moderate-to-strong positive association; suggests hematologic status is informative.
Age	0.38	Moderate positive association with serum vitamin D in this cohort.
Marital status	−0.35	Moderate negative association; may proxy lifestyle or exposure-related differences.
Triglycerides (TG)	0.32	Meaningful positive association among lipid-related variables.
Total cholesterol (TC)	0.24	Weak-to-moderate positive association.
WBC	0.21	Weak positive association.
LDL	0.17	Weak positive association.
HDL	−0.14	Weak negative association.
Genotype indicators	|r| ≤ 0.087	Only weak linear association with vitamin D was observed in this cohort.

**Table 4:** Condensed summary of Wilcoxon signed-rank comparisons between models.

**Pairwise comparison**	**Wilcoxon p-value**	**Interpretation**
SVM vs. EN, LSR, LR, ANN, RAN, RF, RR	3.54 × 10^-3	SVM was significantly different from all other models and showed the strongest overall performance.
RF vs. EN, LSR, LR, ANN, RAN, RR	3.54 × 10^-3	RF was also significantly stronger than the remaining non-SVM methods.
ANN vs. EN, LSR, LR	3.56–3.57 × 10^-3	ANN differed significantly from the linear baselines.
RAN vs. EN, LSR, LR	2.21–2.92 × 10^-2	RANSAC differed significantly from some linear models, although with weaker evidence.
EN vs. LSR	4.44 × 10^-1	No statistically significant difference.
LR vs. LSR	5.89 × 10^-1	No statistically significant difference.
EN vs. LR	2.66 × 10^-1	No statistically significant difference.

### 3.2 Model performance across 30 repeated runs

**Fig. 3:**
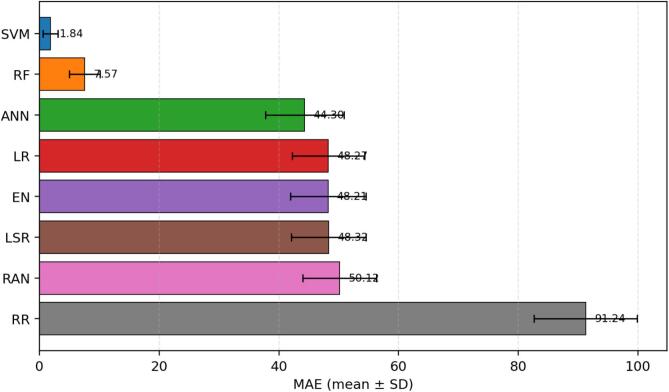
Mean absolute error (MAE) across 30 repeated runs, shown as mean ± standard deviation. Lower values indicate more accurate predictions. SVM achieved the lowest average MAE (1.841 ± 1.261), followed by RF (7.571 ± 2.581), whereas RR showed the largest average MAE (91.241 ± 8.601).

**Fig. 4:**
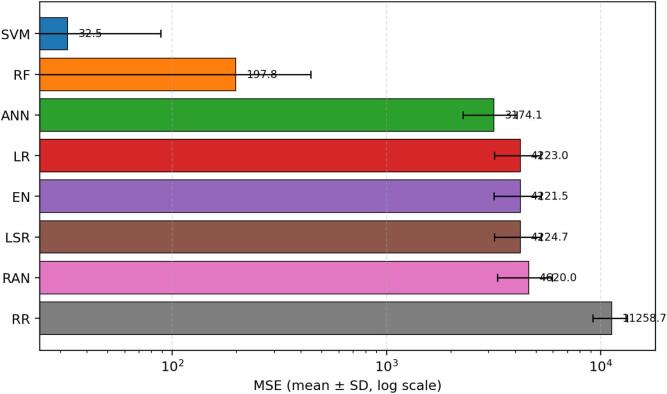
Mean squared error (MSE) across 30 repeated runs, shown as mean ± standard deviation on a logarithmic scale. Lower values indicate better fit, and the log scale is used to accommodate the wide range of error magnitudes across models. SVM and RF again dominate the comparison.

**Fig. 5:**
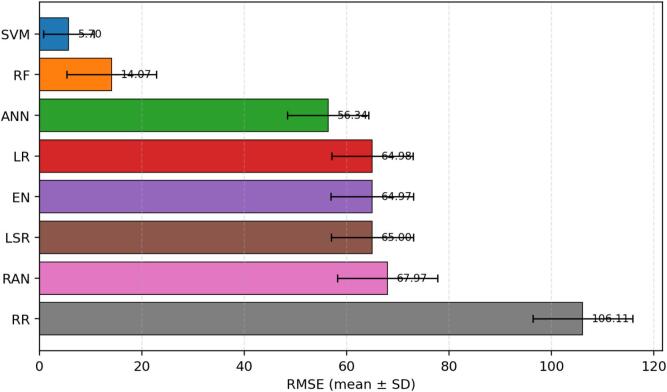
Root mean squared error (RMSE) across 30 repeated runs, shown as mean ± standard deviation. RMSE preserves the unit of the vitamin D outcome, thereby facilitating practical interpretation of the expected prediction error. SVM has the lowest RMSE, followed by RF.

**Fig. 6:**
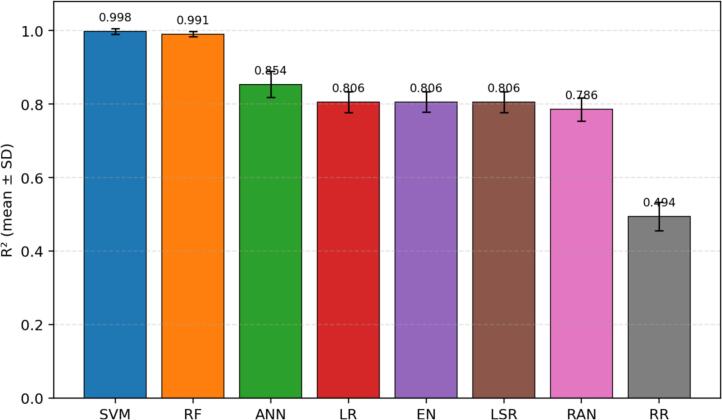
R^2^ across 30 repeated runs, shown as mean ± standard deviation. Higher values indicate that more variance in serum vitamin D is explained by the model. SVM (mean R^2^ = 0.9981) and RF (mean R^2^ = 0.9908) demonstrate near-complete explanatory fit, ANN is intermediate (0.8538), and RR is the weakest (0.4945).

Fig. 3, Fig. 4, Fig. 5, Fig. 6 report the average performance of each model over 30 repeated runs together with standard-deviation error bars. This presentation addresses the reviewer request for uncertainty visualization and makes the comparison between algorithms more informative than single-value reporting.

The MAE comparison indicates a clear hierarchy in absolute prediction error. SVM is the best-performing model, with an average MAE of 1.841 and low variability (SD = 1.261), which indicates both accuracy and stability. RF ranks second with an average MAE of 7.571. ANN occupies an intermediate position (44.301), while the linear baselines LR, EN, and LSR cluster around 48. RR performs worst, suggesting that this regularization setting does not fit the present cohort well.

The MSE results reinforce the MAE ranking. SVM attains the lowest average MSE (32.502), which means that large deviations are strongly suppressed. RF remains the second-best model with an average MSE of 197.832. ANN is notably worse than SVM and RF but still clearly outperforms the linear baselines and RR. Because MSE penalizes large errors more strongly than MAE, the separation between the top models and the weaker models becomes even more evident in Fig. 4.

RMSE leads to the same practical conclusion. SVM produces the smallest average RMSE (5.701), followed by RF (14.065). ANN remains intermediate (56.338), whereas LR, EN, and LSR show substantially larger error spread at approximately 65. RR again performs worst. The convergence of MAE, MSE, and RMSE evidence indicates that SVM is not only the best in a single metric but the strongest model overall for this cohort.

The R^2^ analysis confirms the dominance of SVM and RF. SVM explains nearly all observed variance in the outcome (mean R^2^ = 0.9981), followed closely by RF (0.9908). ANN shows good but clearly lower explanatory power (0.8538). LR, EN, and LSR form a similar middle tier around 0.806, whereas RANSAC is modest at 0.786. RR is substantially weaker at 0.4945, indicating that it captures less than half of the outcome variance in the present setting.

### 3.3 Summary comparison and pairwise significance

**Fig. 7:**
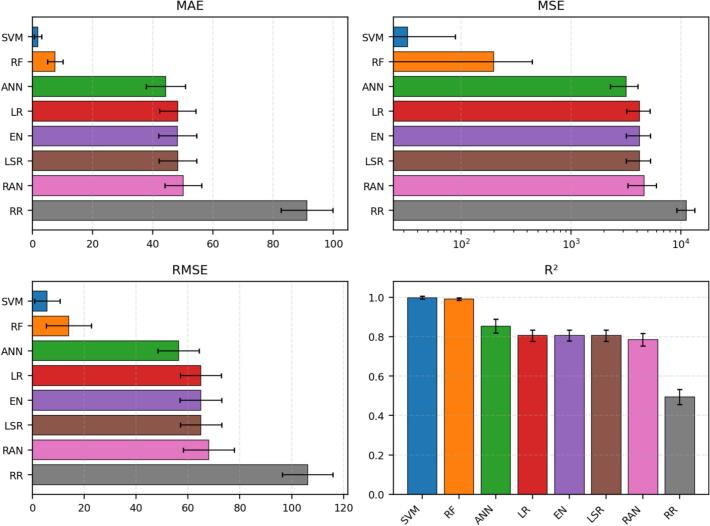
Summary of average performance metrics across the eight developed models. The four panels provide a unified view of MAE, MSE, RMSE, and R^2^, allowing direct comparison between absolute error, squared error, error in original outcome units, and explained variance. Error bars correspond to the standard deviation over 30 repeated runs.

Fig. 7 synthesizes the multi-metric comparison and makes the model ranking visually straightforward: SVM is best across all four metrics, RF is consistently second, ANN is intermediate, LR/EN/LSR occupy a similar middle band, RANSAC is slightly weaker, and RR performs worst. This corrected interpretation replaces the inconsistent statements in the original manuscript and aligns the narrative with the reported performance indicators.

The Wilcoxon results support the descriptive ranking. SVM differs significantly from all other models (p = 3.54 × 10^-3) and therefore stands out as the most reliable estimator in this cohort. RF is also significantly stronger than the remaining non-SVM methods. By contrast, EN, LR, and LSR do not differ significantly from one another, which suggests that the three linear formulations offer broadly similar predictive behavior under the current data conditions.

### 3.4 Discussion

The corrected results show that nonlinear models are more suitable than the regularized linear alternatives for this cohort. SVM achieves the strongest performance, most likely because it can map nonlinear relationships between demographic, hematologic, and lipid features and serum vitamin D. RF also performs exceptionally well, consistent with its ability to capture interactions and nonlinear thresholds while remaining robust to noisy predictors.

From an explainability perspective, the feature-attribute analysis suggests that demographic and basic laboratory variables carry more useful information than the genotype indicators retained in the current dataset. Gender, hemoglobin, age, marital status, and triglycerides emerged as the most informative attributes. This finding is practically relevant because these variables are typically easier to obtain than specialized molecular markers.

Several limitations should be acknowledged. The sample size is modest, the cohort is study-specific, and external validation was not available in the current source file. Therefore, the strong performance of SVM and RF should not be interpreted as universal superiority across all populations. In addition, the interpretability layer added here is based on transparent feature-attribute analysis from the correlation structure and model families rather than full post-hoc instance-level explainers. A future extension should apply model-specific explainability methods such as SHAP [21] on a larger externally validated dataset.

## 4 Conclusion

This revised study compared eight regression algorithms for estimating serum vitamin D level as a support tool for vitamin D deficiency assessment. Across 30 repeated runs, SVM was the strongest model overall, followed by RF, while ANN showed moderate performance and RR was the weakest. The revised abstract, results, and conclusion now consistently reflect the quantitative indicators reported in the manuscript. In response to reviewer comments, the paper now also includes an explainability-oriented feature-attribute analysis, detailed mathematical formulations, richer figure legends, and error bars for Fig. 3, Fig. 4, Fig. 5, Fig. 6, Fig. 7. Overall, the results suggest that well-selected nonlinear models can support cost-conscious vitamin D assessment workflows, but larger multi-center cohorts and external validation are still needed before clinical deployment.

**Data Availability Statement**

Dataset 132 is a study-specific, de-identified cohort dataset used for the present analysis. It is not a public benchmark dataset. Subject to institutional approval and data-protection requirements, the dataset may be made available by the corresponding author on reasonable request.

## CRediT authorship contribution statement

**Ahmad Al-Qerem:** . **Ruzayn Quddoura:** Funding acquisition. **Abdulhakeem Issa:** . **Asma Sbaih:** Investigation, Formal analysis, Data curation.
